# Necrotizing Fasciitis Following Wet Cupping: A Case Report

**DOI:** 10.7759/cureus.14039

**Published:** 2021-03-22

**Authors:** Turki Alajmi, Abdulmalek Aljulaihim, Mosa Alzahrani, Saad Aljuhayyiam

**Affiliations:** 1 Orthopedic Surgery, Prince Mohammed Bin Abdulaziz Hospital, Riyadh, SAU; 2 Trauma, Prince Mohammed Bin Abdulaziz Hospital, Riyadh, SAU

**Keywords:** necrotizing fasciitis, wet cupping, pseudomonas

## Abstract

Cupping therapy is a widely practiced form of adjunctive medicine and it has been used since ancient times. It involves using cups over the skin to create negative pressure. The exact mechanism by which cupping therapy exerts its effects is unknown, but it is thought to act as an artificial kidney. In this report, we present a case of a 35-year-old male who developed *Pseudomonas-*positive necrotizing fasciitis following wet cupping therapy. He refused surgical intervention and subsequently died. Necrotizing fasciitis is a severe soft tissue infection that has a high mortality rate. The only proven intervention to improve survival is aggressive surgical debridement. There have been a few reports of infectious complications following wet cupping, including lumbar abscess and septic arthritis; however, to the best of our knowledge, this is the first report of a necrotizing soft tissue infection following cupping therapy.

## Introduction

Cupping therapy is a technique that uses cups placed over the skin to create a negative pressure through suctioning. It can be further classified into two types: wet and dry cupping [1]. Dry cupping is relatively benign, noninvasive, and does not cause bleeding. However, wet cupping involves bloodletting, and it also involves creating cuts through the skin using blades or pins and using the negative pressure to remove the blood [2]. This ancient practice goes as far back as Herodotus and Hippocrates, when people used it to treat many pathologies including back pain, gynecological complaints, and extremity illnesses [3]. The exact mechanism by which cupping therapy exerts its presumably therapeutic effects is unknown, but one theory called Tiabah suggests that wet cupping mimics an artificial kidney; while kidneys in vivo filter hydrophobic material through the glomeruli utilizing normal pressure filtration, wet cupping filters both hydrophobic and hydrophilic material by utilizing high-pressure filtration [4].

Cupping therapy is generally considered safe with infrequent adverse effects that range from mild to moderate in severity, with few reports of severe or life-threatening complications [5]. Non-preventable complications of cupping include headaches, vasovagal attacks, and the Koebner phenomenon, and preventable complications include anemia, scars, bullae formation, and superficial skin irritation and infection [5]. Risk of infection, scarring, and vasovagal attacks along with overall complications are seen more frequently in wet cupping.

In this report, we present a case of bilateral necrotizing fasciitis of the lower extremity caused by wet cupping. To the best of our knowledge, based on a review of the literature, this is the first case report of necrotizing fasciitis following wet cupping.

## Case presentation

A 35-year-old healthy male presented to our emergency room with bilateral lower extremity swelling for two weeks, for which he had already undergone wet cupping (Hijama) seven days prior; he had subsequently developed severe bilateral lower extremity pain, redness, skin sloughing, and increase in swelling. The systemic review revealed that the patient had a history of chills and night sweats that had started with his symptoms. The patient denied any other complaints, and his family also denied any history of psychiatric illnesses, smoking, or drug abuse. Furthermore, he was a full-time university business professor, who was single and living alone.

**Figure 1:**
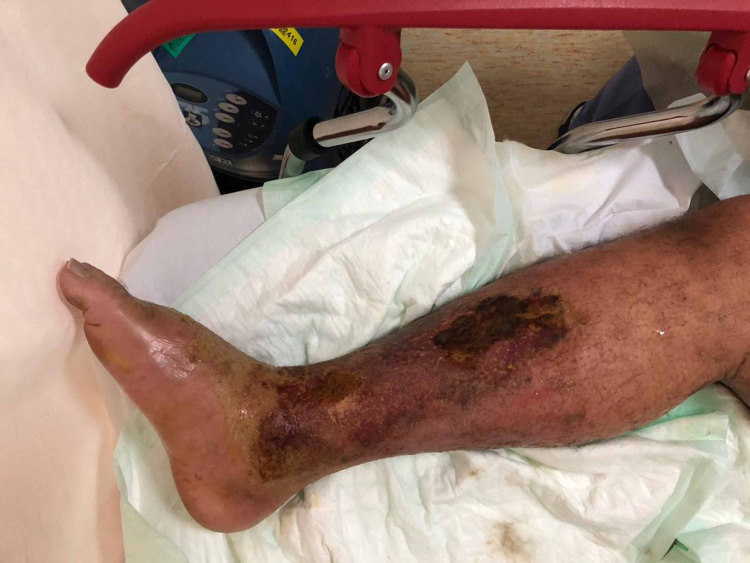
Patient's right lower limb upon presentation

**Figure 2:**
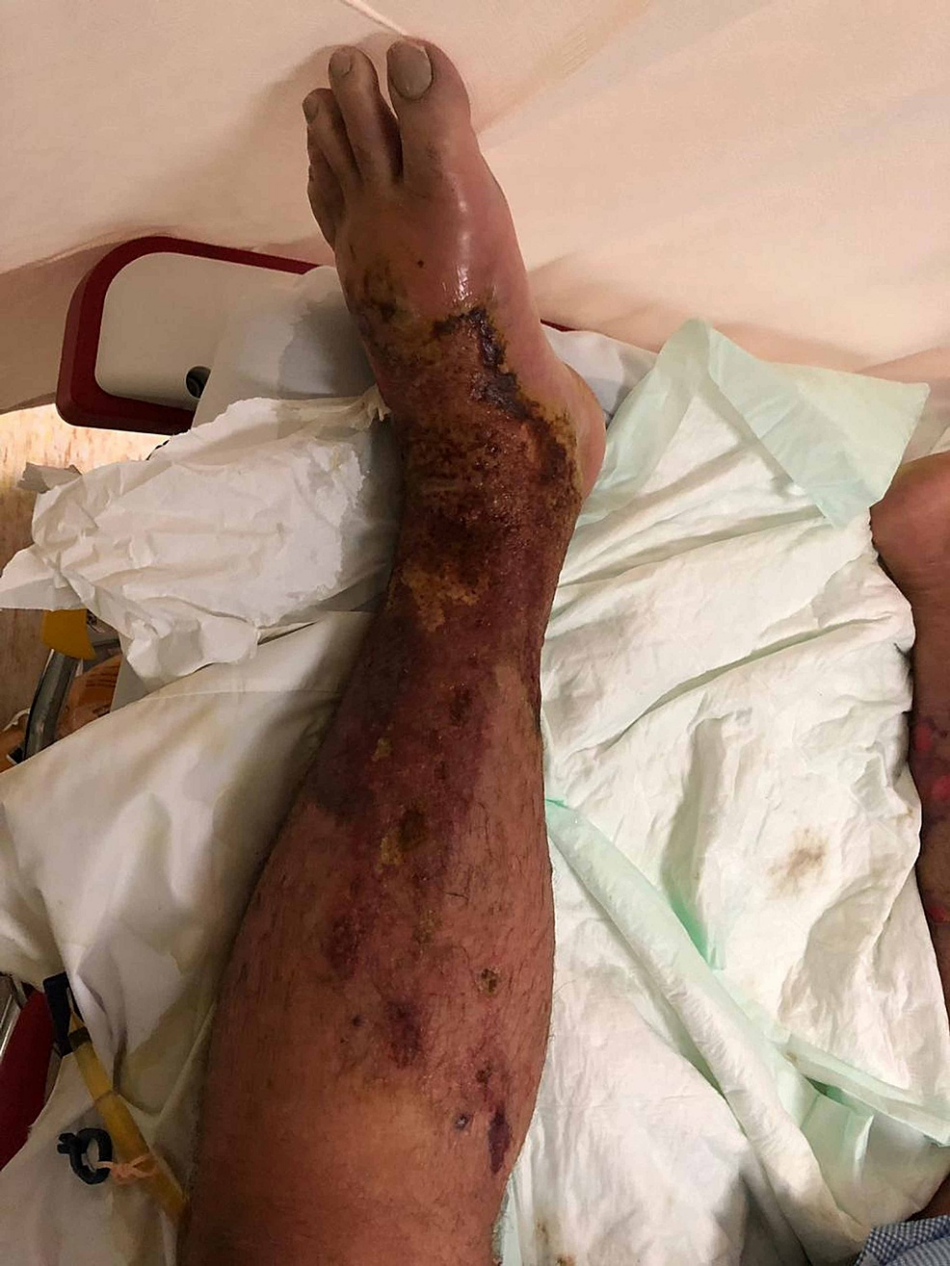
Patient's left lower limb upon presentation

The patient was febrile at 39 °C, had a BP of 100/60 mmHg, BMI of 18 kg/m^2^, and oxygen saturation of 97% on room air; his Glasgow Coma Scale (GCS) was 14/15. He had bilateral lower limb swelling and bullae along with pitting edema and skin sloughing (Figures 1, 2).

The lower limbs were extremely tender to palpitation, and a range of motion test could not be attempted due to the severe pain. Bilaterally, his femoral pulses were palpable; however, his popliteal pulses were nonpalpable but audible by Doppler. Furthermore, his posterior tibialis and dorsalis pedis pulses were neither palpable nor audible by Doppler. CT angiogram of bilateral lower extremities showed patent arterial systems with no extravasation, diffuse skin thickening with subcutaneous edema, extensive areas of intramuscular collections, and fat stranding reaching the popliteal fossa. Blood and wound cultures were obtained along with a complete septic workup of the patient, which came back positive for *Pseudomonas* species. His labs in the emergency room are shown in Table 1.

**Table 1:** Patient's labs on presentation WBC: white blood cells; Hgb: hemoglobin; CRP: C-reactive protein

Parameter	Result
WBC, /mL	85,000
Hgb, g/dl	12
Sodium, mmol/L	131
Creatinine, mg/dL	3.26
CRP, mg/L	221
Glucose, mg/dL	200

The patient was admitted with a diagnosis of bilateral lower extremities necrotizing fasciitis based on his clinical presentation and Laboratory Risk Indicator for Necrotizing Fasciitis (LRINEC) score of 12.

**Figure 3:**
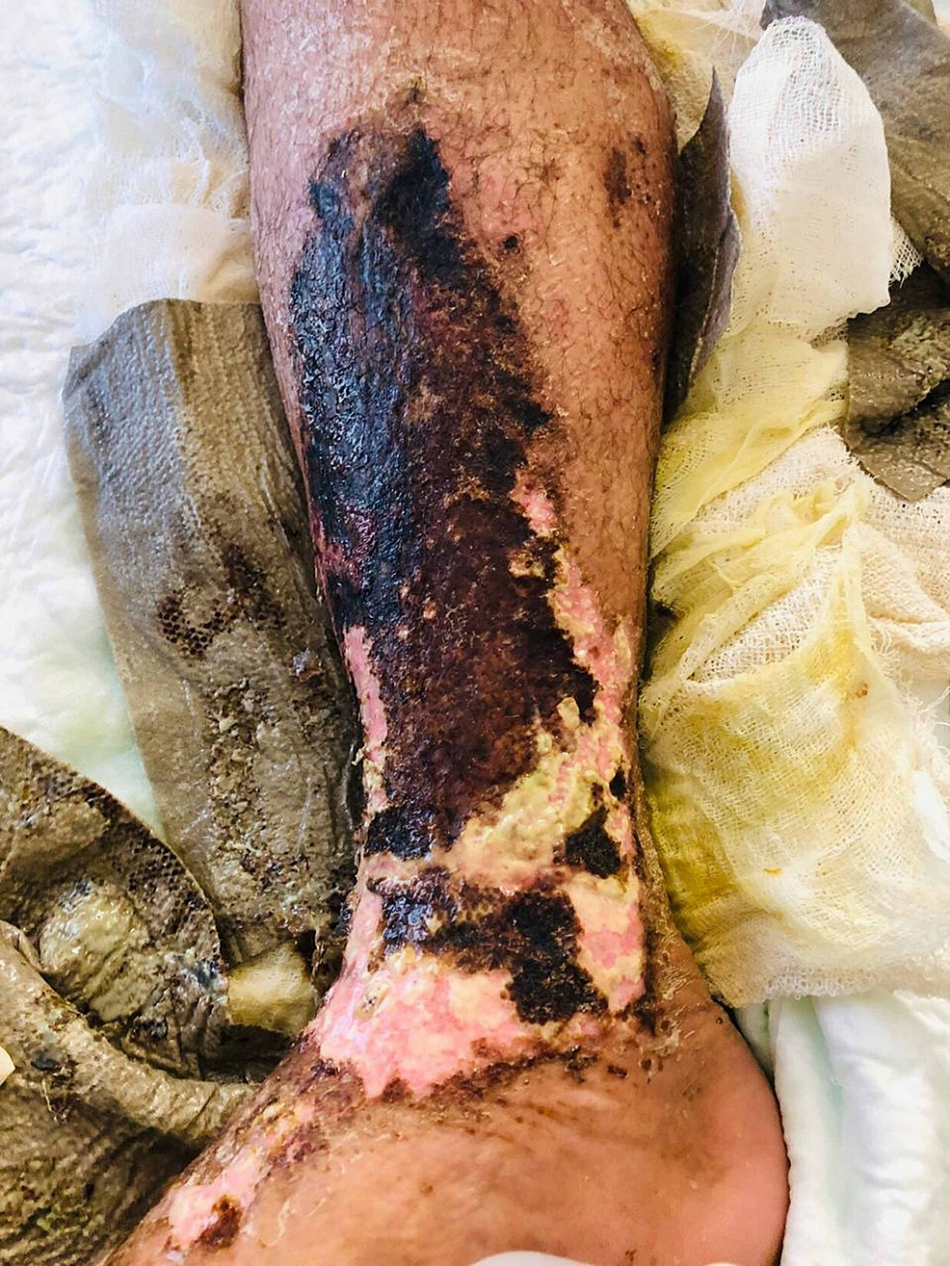
Patient's right lower limb one week after presentation

**Figure 4:**
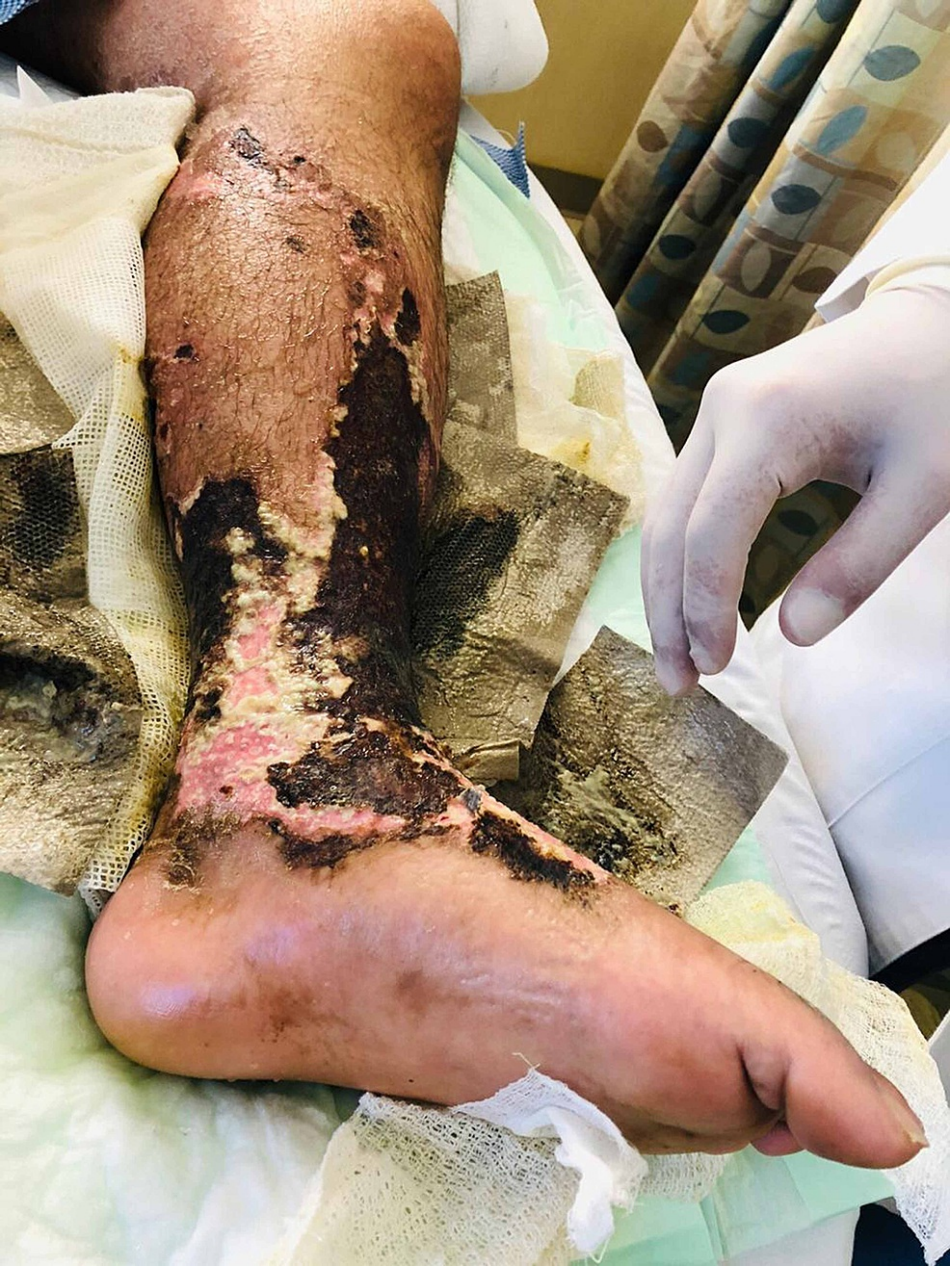
Patient's left lower limb one week after presentation

As it was a surgical emergency and based on his toxic clinical status and examination finding along with his labs, the optimal management as decided by the orthopedic surgery department was bilateral above-knee amputation versus aggressive debridement, which the patient refused completely. Plastic, vascular, and general surgery services were urgently consulted, and they recommended urgent surgical intervention; unfortunately, the patient kept on refusing such life-saving options. Psychiatric and legal department opinion was also sought, but both concluded that the patient was competent enough to make his own decisions. The patient refused any form of surgical intervention including irrigation and debridement. The patient was admitted under the care of internal medicine and was started on vancomycin and meropenem in order to cover for methicillin-resistant *Staphylococcus aureus* (MRSA) and *Pseudomonas* species due to the penetrating nature of wet cupping; conversion to meropenem as monotherapy was carried out based on his culture results. Within the following week, the patient developed hemodynamic instability and was intubated and mechanically ventilated in the Intensive care unit, and his lower limbs had acquired an unsightly appearance by that time, as shown in Figures 3, 4.

**Figure 5:**
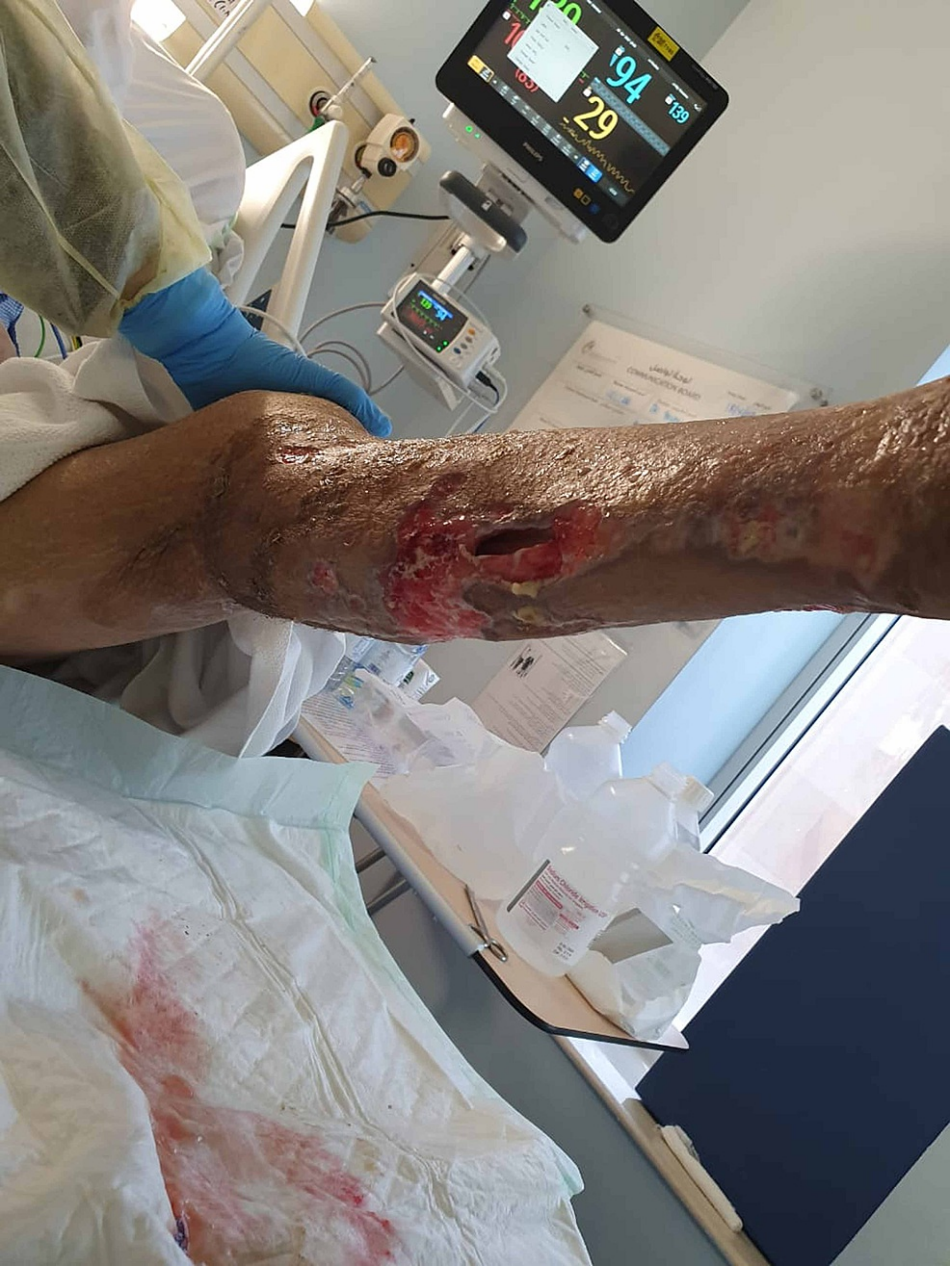
Lower limb soft tissue defects - image 1

**Figure 6:**
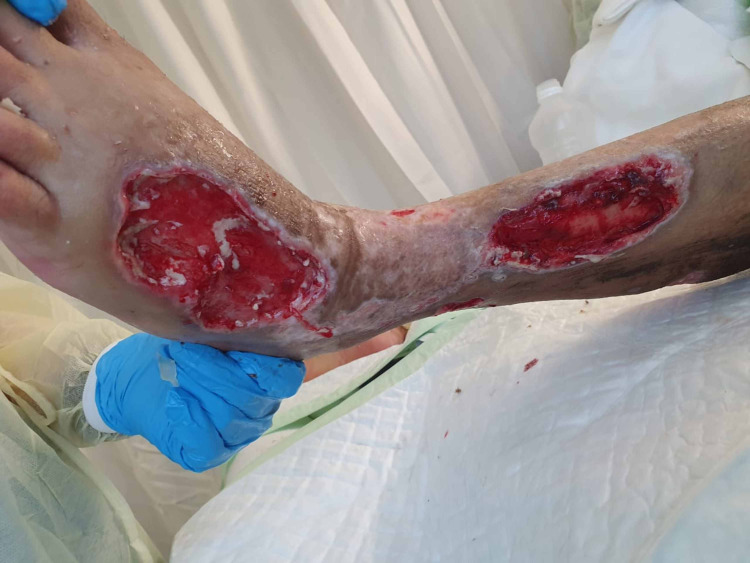
Lower limb soft tissue defects - image 2

Two weeks later, the patient was extubated and his condition improved, and one month later, his labs improved and his pain subsided. Hence, the decision to discharge the patient was taken since all treatment options had been exhausted. By the time of his discharge, the patient had completely lost his lower limb sensation and function and had developed multiple soft tissue defects (Figures 5, 6). The plastic surgery department had decided that the soft tissue bed was contaminated and could not be rectified without formal debridement in the OR. However, the patient refused this option since he was informed that there was a possibility that he might lose his limbs based on the intraoperative decision. On the day of discharge, the patient developed shortness of breath and hypoxia; an urgent CT scan was carried out and he was found to have a massive pulmonary embolism, which eventually led to his death despite all resuscitative measures.

## Discussion

Although wet cupping is considered to be a relatively safe form of adjunctive medical procedure, it can occasionally lead to life-threatening complications. Necrotizing soft tissue infections form part of a distinct spectrum of infections that have a mortality rate affecting one-third of the patients and average mortality of over 20% [6]. Necrotizing fasciitis is further classified into various types based on the causative organism isolated on Gram stain and culture: type 1, which constitutes 80-90% of the infections, is polymicrobial and caused by non-Group A S*treptococcus* along with aerobes or possibly anaerobes; type 2 is defined based on the presence of Group A-beta hemolytic *Streptococci;* type 3 is caused by marine *Vibrio* bacteria, which are gram-negative rods; and type 4, which is extremely rare, is caused by fungal infections, mainly *Aspergillus*, Zygomycetes, *Candida, Mucor,* and *Rhizopus* species [7]. Type 1 necrotizing fasciitis is usually found in postoperative perianal or abdominal infections, which are characteristic of immunosuppressed patients, and it is thought to result from synergistic bacteria. Type 2 is considered the “flesh-eating” infection and develops within the extremities of healthy patients. Type 3 can result from a simple puncture wound or insect bite that is then exposed to marine animals or seawater. Type 4 can be found in immunocompetent individuals following severe trauma and burns, with the exception of *Candida* species-induced kind, which has a predilection for immunosuppressed patients (Table 2) [8].

**Table 2:** Classification of necrotizing fasciitis GABHS: group A beta-hemolytic *Streptococci*

Type	Organism	Characteristics
1	Polymicrobial (non-GABHS) Gram-positive: *Enterococci*, *Staphylococcus aureus*, Coagulase-negative *Staphylococci;* Gram-negative: *Escherichia coli*, *Pseudomonas*, *Klebsiella;* Anaerobic: *Bacteroides* spp, *Clostridium* spp	Most common; associated with post-abdominal and perianal surgery; seen in immunosuppressed patients
2	Group A beta-hemolytic *Streptococci*	Second most common; seen in healthy individuals; may occur without preceding trauma; seen in limbs
3	Marine *Vibrio* spp: *Vibrio vulnificus*, *Vibrio damsela*, *Vibrio parahaemolyticus*	After insect bite or puncture wound followed by seawater exposure
4	*Aspergillus*, Zygomycetes, *Candida, Mucor, Rhizopus* species	Seen in immunocompetent individuals, after severe trauma and burns; *Candida* species seen in immunosuppressed patients

The diagnosis of necrotizing fasciitis is primarily clinical, as no test can be as valuable as a very high index of suspicion, and the average diagnosis usually takes three days and may even be delayed for weeks [9]. However, in 2004, the LRINEC method was developed, which has shown a 92% positive predictive value and a 96% negative predictive value when the score 6 was used as a cutoff to determine the presence of necrotizing soft tissue infections (Table 3) [10]. The major indicator for the diagnosis of necrotizing fasciitis is the presence of necrosis of the facial planes during surgery. Imaging may aid in the diagnosis as well: X-rays may show gas within the soft tissue, and CT imaging is particularly useful in pediatric patients as thickening of the soft tissues and fat stranding is present in over 80% of the cases [9].

**Table 3:** Laboratory Risk Indicator for Necrotizing Fasciitis (LRINEC) scoring parameters CRP: C-reactive protein; WBC: white blood cells; Hgb: hemoglobin

Parameter	Value		Score
CRP, mg/L	>150		0
	<150		4
WBC count, /mL	<15,000		0
	15,000–20,0000		1
	>25,000		2
Hgb level, g/dl	>13.5		0
	11–13.5		1
	<11		2
Sodium level, mmol/L	>135		0
	<135		2
Creatinine level, mg/dL	<1.6		0
	>1.6		2
Glucose level, mg/dL	<180		0
	>180		1
Score		Probability of necrotizing fasciitis	
5 or less		<50%	
6–7		50–75%	
8 or more		>75%	

On histopathology, early findings routinely show extensive vessel thrombosis, necrosis of the fascia, nerves, and fat [11]. Later in the disease process, there is usually a dense inflammatory infiltrate with a predominance for neutrophils, and liquefactive necrosis that usually results in positive tissue cultures [12].

**Figure 7:**
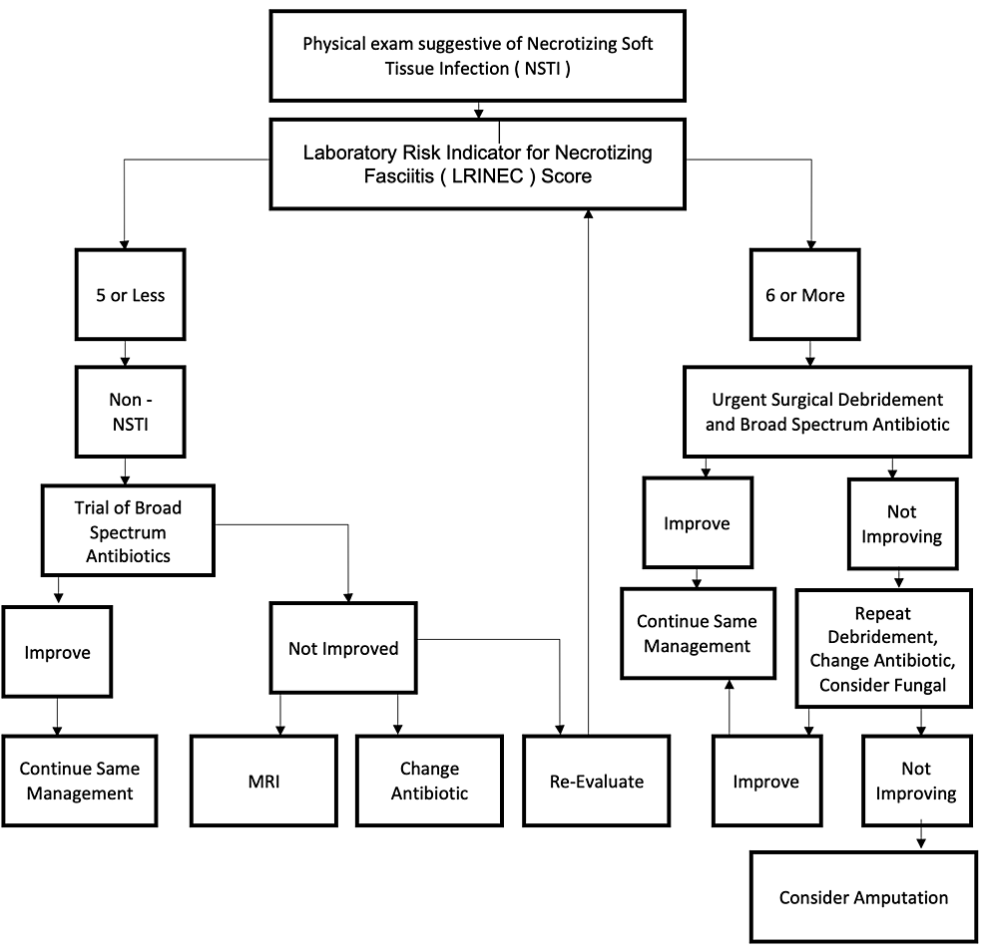
Treatment algorithm

Aggressive surgical debridement is the predominant treatment protocol as it is the single most effective method for reducing mortality rates [13]; surgical debridement should not be delayed for imaging purposes as it is necessary to remove all necrotic tissue and reduce bacterial load in order to give the immune system a chance to fight the disease [14]. Adjunctive therapies along with surgical debridement and broad-spectrum antibiotics have been proven to be effective, and these help with adequate fluid resuscitation, restoration of protein deficiencies, and maintaining electrolytes balances as these patients usually present with sepsis or septic shock. A new form of adjunct therapy involves administering systemic intravenous immunoglobulin, which in theory can blunt the host's immune response to bacterial toxins; however, data have shown conflicting results [14]. A proposed algorithm of treatment is shown in Figure 7.

Our review of literature elicited a few reports about serious infectious complications following wet cupping, but none of them involved a condition as severe as necrotizing fasciitis [15,16]. One study has reported the case of a patient who developed a lumbar spinal abscess, which necessitated surgical debridement [16], and another report has discussed a patient who developed septic arthritis of the knee [15]. However, there has been a growing body of evidence suggesting that therapy using wet cupping can be a source of blood-borne infections including hepatitis C [17]. Herpes simplex virus (HSV) infection of the skin has also been reported secondary to cupping therapies [18].

## Conclusions

Cupping therapy is a widely used form of adjunctive medical procedure. Although proven to be effective, it may cause serious complications as demonstrated in our case. Necrotizing fasciitis is a severe soft tissue infection that necessities an extremely high index of suspicion for diagnosis, and surgical management should not be delayed as it is the only treatment that has been shown to be effective in increasing survival rates. Furthermore, drastic measures should be taken and all options should be exhausted in order to save a patient's life, which may include psychiatric help, family counseling in order to convince the patient to undergo the appropriate treatment, and even seeking the service of legal authorities.
